# Molecular epidemiological characteristics of echovirus 6 in mainland China: extensive circulation of genotype F from 2007 to 2018

**DOI:** 10.1007/s00705-020-04934-7

**Published:** 2021-02-26

**Authors:** Wenjun Cheng, Tianjiao Ji, Shuaifeng Zhou, Yong Shi, Lili Jiang, Yong Zhang, Dongmei Yan, Qian Yang, Yang Song, Ru Cai, Wenbo Xu

**Affiliations:** 1 Medical School, Anhui University of Science and Technology, Huainan, 232001 Anhui People’s Republic of China; 2 NHC Key Laboratory of Medical Virology and Viral Diseases, National Institute for Viral Disease Control and Prevention, Chinese Center for Disease Control and Prevention, Beijing, People’s Republic of China; 3 Hunan Provincial Centers for Disease Control and Prevention, Changsha, People’s Republic of China; 4 Jiangxi Provincial Centers for Disease Control and Prevention, Nanchang, People’s Republic of China; 5 Yunnan Provincial Centers for Disease Control and Prevention, Kunming, People’s Republic of China

## Abstract

Echovirus 6 (E6) is associated with various clinical diseases and is frequently detected in environmental sewage. Despite its high prevalence in humans and the environment, little is known about its molecular phylogeography in mainland China. In this study, 114 of 21,539 (0.53%) clinical specimens from hand, foot, and mouth disease (HFMD) cases collected between 2007 and 2018 were positive for E6. The complete VP1 sequences of 87 representative E6 strains, including 24 strains from this study, were used to investigate the evolutionary genetic characteristics and geographical spread of E6 strains. Phylogenetic analysis based on VP1 nucleotide sequence divergence showed that, globally, E6 strains can be grouped into six genotypes, designated A to F. Chinese E6 strains collected between 1988 and 2018 were found to belong to genotypes C, E, and F, with genotype F being predominant from 2007 to 2018. There was no significant difference in the geographical distribution of each genotype. The evolutionary rate of E6 was estimated to be 3.631 × 10^-3^ substitutions site^-1^ year^-1^ (95% highest posterior density [HPD]: 3.2406 × 10^-3^-4.031 × 10^-3^ substitutions site^-1^ year^-1^) by Bayesian MCMC analysis. The most recent common ancestor of the E6 genotypes was traced back to 1863, whereas their common ancestor in China was traced back to around 1962. A small genetic shift was detected in the Chinese E6 population size in 2009 according to Bayesian skyline analysis, which indicated that there might have been an epidemic around that year.

**Supplementary Information:**

The online version contains supplementary material available at 10.1007/s00705-020-04934-7.

## Introduction

Enteroviruses (EVs) are members of the genus *Enterovirus* in the family *Picornaviridae* and order *Picornavirales*. There are currently over 100 recognized EV types that fall into four main species (designated A to D) based on phylogenetic analysis [1]. Echovirus 6 (E6), a member of the species *Enterovirus B*, is a common virus that causes a wide range of diseases in humans. Although infection in immunocompetent individuals is often asymptomatic or induces mild fever, E6 is a common etiological agent in nervous system diseases, including aseptic meningitis (AM), encephalitis, and acute flaccid paralysis (AFP) [2–5], and it can cause persistent and/or widely disseminated systemic infection in immunosuppressed individuals and neonates [6–9].

In epidemics associated with these diseases, E6 has been the main enterovirus type in Europe. In 2006-2007, E6 (40.74%) was the most frequently isolated enterovirus type in Greece and was divided into three clades in a phylogenetic tree [10]. Moreover, E6 was one of the most common pathogens (23%) associated with AM in France in 2006 [11]. In 2011, environmental samples of raw sewage were collected in Poland, and E6 was the fourth most commonly identified enterovirus (15%). Since then, a high level of E6 activity has been observed in Poland. In 2012, E6 was responsible for a major outbreak of AM in central-eastern Poland [12, 13]. E6 is also widespread in America and Asia. According to enterovirus surveillance in the United States from 1970 to 2005, E6 was the main pathogen among infants approximately 1 year old [14]. In Brazil, an outbreak of AM in early 2004 was related to the high activity of E6, and E6 was one of the most common viruses (66.8%) detected from 2013 to 2017 [15].

With the widespread application and acceptance of molecular genotyping methods based on the entire VP1 sequence [16], countries around the world are currently monitoring E6. Many researchers have proposed different E6 classifications of genotypes based on full-length VP1 sequences. In Korea, E6 (33.1%) was detected in AM cases in 2008 and divided into three genetic groups, A, B, and C [17]. From April to July 2005, an outbreak of AM caused by E6 occurred in Anhui Province, China, and the strains were divided into clusters A, B, and C [18]; in 2010, E6 in Yunnan Province was divided into five distinct groups [19]. In 2012, researchers in Japan also found E6 to form three main clusters (A, B, and C) [20]. However, most studies on E6 have been limited to a certain geographical area or based on the monitoring of environmental sewage [21–24], whereas little is known about its transmission networks and geographical distribution. This study covered global E6 sequences and further classified E6 based on previous research.

## Materials and methods

### Specimen collection and virus isolates

A total of 22,690 clinical specimens collected between 2007 and 2018 were tested for EV using a commercial real time RT-PCR kit (Shuoshi Biotech, Jiangsu, China) in a nationwide HFMD surveillance network in mainland China, and 21,539 positive samples were inoculated onto RD cells, which were obtained from the WHO Global Specialized Poliovirus Laboratory at the US CDC. A cytopathic effect (CPE) was observed and within one week after the cells were infected, and samples were collected and frozen (-80 °C) for long-term storage. Viruses in the cell supernatants were sequenced, and 114 (0.53%) of the samples were positive for E6 (Supplementary Table 1).

Twenty-four sequences were selected according to specimen quality, time since isolation, and geographical location for 2009-2018, encompassing 12 of 31 provinces in China, and investigated. Among the 24 cases represented by these samples, seven were identified as severe, and one patient died. (Supplementary Table 2)

### Determination of the full-length VP1 sequence of E6

Nucleic acid was extracted using a MagaBio plus Virus RNA Purification Kit (product number: BSC58S2DB China), and the region encoding the E6 capsid protein VP1 was amplified by reverse transcription polymerase chain reaction (RT-PCR) [25]. The cycling conditions used were as follows: 50 °C for 30 min; 94 °C for 3 min; 32 cycles at 94 °C for 30 s, 50 °C for 30 s, and 72 °C for 1 min and 20 s; and a final extension step at 72 °C for 10 min. The PCR products were sequenced using capillary electrophoresis in a 3730xl-DNA Analyzer. Sequences were edited and assembled using Sequencher 5.0 software (Gene Codes, Ann Arbor, MI, USA). The sequences were deposited in the GenBank database under accession numbers MT108421 to MT108430.

### Dataset construction of global and Chinese E6 VP1 sequences

A total of 708 full-length sequences of VP1 were retrieved from GenBank up to September 6, 2019. These sequences were from viruses isolated from various countries around the world from 1955 to 2017. These sequences contain a variety of source information, including AM/meningitis, AFP, HFMD, and environmental monitoring (Supplementary Table 3). Sequences with a lack of source information, low quality, or high similarity to other sequences were eliminated, and a total of 63 representative sequences from GenBank, together with 24 selected sequences from this study were used for phylogenetic analysis (Supplementary Table 2). We selected 84 of a total of 87 representative sequences based on the collection data to describe the evolutionary origin of E6 on a global scale.

### Phylogenetic and evolutionary origin analysis

A total of 87 sequences were aligned using Clustal W in MEGA 5.0 and used to construct a maximum-likelihood (ML) tree [26] based on the 867-bp full-length VP1 sequence with 1000 bootstrap replicates. Sequences that differed by at least 15% were assigned to different genotypes [27].

Eighty-four full-length sequences of VP1 with collection date information were aligned in MEGA (v5.0), and we chose TN93 (Tamura-Nei) + G (γ) as the best model. The Bayesian Markov chain Monte Carlo (MCMC) method in BEAST (v1.7.5) was used to estimate the rate of evolution and the temporal phylogenies of the VP1 nucleotide sequences [28, 29]. The sequences were analyzed using a strict clock, with Tracer (http://beast.bio.ed.ac.uk/Tracer) to check the convergence of chains and estimate the effective sample size. The MCMC run consisted of 5 × 10^7^ generations, and the sampling frequency was set to 5000 generations. A maximum-clade-credibility (MCC) tree was constructed using Tree Annotator v1.7.5. The first 10 percent of the steps from each run were removed as burn-in, and the results were visualized using FigTree v.1.4.2 (http://tree.bio.ed.ac.uk/software/). According to the coalescent-based inference method, sequence datasets can be used to estimate various population genetic parameters, including population size [30]. The Bayesian skyline analysis method in BEAST software was used to select Chinese E6 sequences of genotypes E and F after 2005 (including 2005) based on the 87 VP1 full-length sequences selected as described above.

## Results

### Identification of six E6 genotypes worldwide by phylogenetic analysis based on complete VP1 sequences

**Fig. 1:**
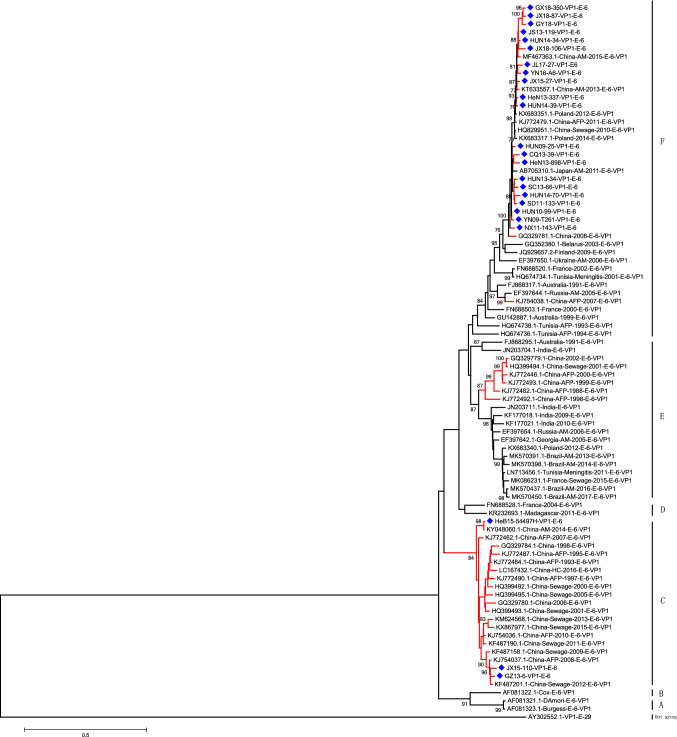
Maximum-likelihood phylogenetic dendrogram based on VP1 sequences (867bp) of 87 representative E6 isolates collected between 1955 and 2018. Six E6 genotypes (A, B, C, D, E and F) were identified. The scale bar represents a genetic distance of 0.5 nucleotide substitutions per site. Full-length sequences of E6 VP1 from China occupy the red branch of the tree, and blue diamonds indicate sequences determined in this study.

According to published enterovirus genotyping criteria, a difference of at least 15% between complete VP1 nucleotide sequences should be used to distinguish genotypes [31]. Six E6 genotypes (A, B, C, D, E and F) were identified by phylogenetic analysis based on complete VP1 sequences (Fig. 1), and the nucleotide sequence divergence among these genotypes ranged from 17.26 to 23.02% (Table 1). Genotype A is composed of the prototype strain D'Amori, and the strain of Cox was classified as genotype B in most studies. All of the sequences belonging to genotype C were isolated in China. Genotype D mostly spread throughout France and Madagascar in 2004 and 2011. Genotypes E and F have spread over Asia, Europe, America and Africa. In our analysis of the epidemic of E6 in China, genotype C was mainly found in sewage. Genotype E was mainly associated with AFP cases, but genotype F was associated with many kinds of diseases, but mainly meningitis (Table 2).

**Table 1:** Estimates of evolutionary divergence of sequence pairs belonging to different genotypes/subgenotypes

Genotype	Nucleotide						Amino acid					
	A	B	C	D	E	F	A	B	C	D	E	F
A												
B	0.1978						0.0460					
C	0.2302	0.2208					0.0534	0.0620				
D	0.2223	0.2186	0.1936				0.0717	0.0699	0.0415			
E	0.2222	0.2159	0.1983	0.1762			0.0711	0.0665	0.0422	0.0278		
F	0.2255	0.2182	0.2006	0.1800	0.1726		0.0732	0.0680	0.0430	0.0257	0.0203

**Table 2:** Information about the 356 Chinese E6 VP1 sequences used in this study

Disease type	Number of cases						Number of sequences (percentage)	Province	Years
	A	B	C	D	E	F			
AM/Meningitis	**-**	-	4	-	-	72	76 (21.35%)	Shandong/Zhejiang/Anhui/Yunnan	2005-2015
HFMD	**-**	-	6	-	2	33	41 (11.52%)	Shandong/Sichuan/Fujian/Yunnan/Hebei/Ningxia/Jilin/Sichuan/Guizhou/Guangxi/Chongqing/Hunan/Henan/Jiangsu/Jiangxi	1998-2018
AFP	**-**		25	-	15	33	73 (20.51%)	Yunnan/Shandong/Guangdong	1988-2014
Sewage	**-**		97	-	1	51	149 (41.85%)	Yunnan/Shandong/Guangdong	2000-2015
Healthy children	**-**	-	1	-	6	10	17 (4.78%)	Yunnan	2013-2016
Total	-	-	133	-	24	199	356	17 provinces	1988-2018

E6, echovirus 6; AM, aseptic meningitis; HFMD, hand, foot, and mouth disease; AFP, acute flaccid paralysis. "-" indicates no cases.

### Prevalence of the E6 strain in mainland China

By collating 708 sequences downloaded from GenBank together with 114 clinical specimens from our laboratory, we found that E6 has become increasingly prevalent, with increasing reports from a growing number of countries after 2008, especially from 2008 to 2013, reaching 45.86% (Supplementary Table 4). At the same time, a total of 356 Chinese sequences from 17 of 31 provinces, were also identified and analyzed (Table 2). Nearly half of them were isolated from sewage, followed in frequency by cases of AM/meningitis and AFP.

### Three genotypes cocirculated in mainland China

All of the E6 strains that circulated in China since 1988 were of genotypes C, E, and F, with most of the isolates (53.61%) belonging to genotype F.

All E6 strains isolated in China were of genotype C and were monitored from 1993 to 2016.

Genotype E has been found since 1988 in Yunnan Province, southwest China, but it was not detected via surveillance after 2002. Genotype F was first isolated in 2007 and continued to circulate until 2018; it was the largest branch with the most extensive geographic distribution (including 12 provinces/cities in China). It is worth noting that all the severe and fatal cases in this study involved genotype F.

Genotype F was widely detected in cases of AM/meningitis, HFMD, and AFP and in samples from sewage and healthy children, with AM/meningitis cases and sewage samples being the most frequent sources. Similarly, genotype C was mostly detected in sewage, based on environmental monitoring. Although genotype E was not isolated from AM/meningitis cases in China, it was prevalent in other countries.

### Evolutionary origin and speculation of population changes in E6 based on the VP1 gene

**Fig. 2:**
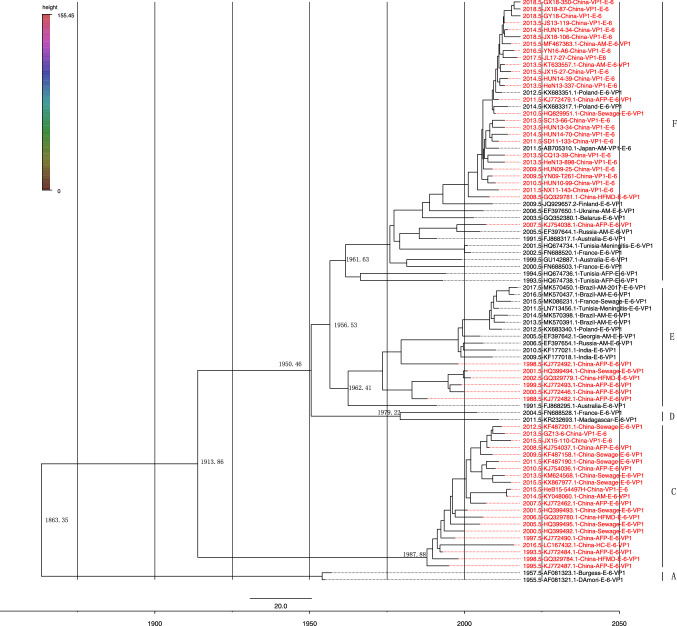
MCMC tree based on 84 complete VP1 sequences of E6. The nucleotide substitution model used was the TN93 (Tamura-Nei) + G(γ) model. The MCMC run consisted of 5 × 10^-7^ generations, and the sampling frequency was set to 5000 generations. The scale bar represents time in years, and the strains isolated in China occupy the red branch of the tree.

**Fig. 3:**
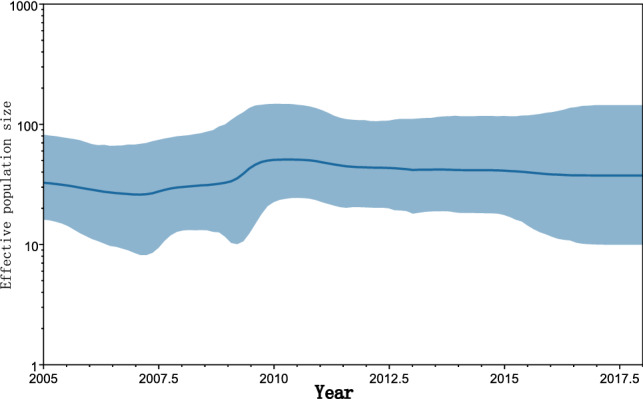
Bayesian skyline plot constructed based on 87 full-length VP1 sequences of Chinese E6 isolates of genotypes E and F in or after 2005. Slight fluctuations occurred in China during the years 2007-2009, especially circa 2009, and the population tended to expand on a small scale.

An MCMC tree based on full-length VP1 sequences of 84 globally distributed E6 strains was generated using BEAST (Fig. 2). The average base substitution rate of E6 worldwide from 1955 to 2018 was 3.631 × 10^-3^ substitutions site^-1^ year^-1^ (95% HPD: 3.2406 × 10^-3^-4.031 × 10^-3^ substitutions site^-1^ year^-1^); thus, the earliest ancestor of E6 can be traced back to 1863 (95% HPD:130-185 years ago). The origin of genotype C was circa 1988 (95% HPD: 28-32 years ago); the origin of genotype E can be traced back to 1962 (95% HPD: 50-62 years ago), which is very close to the date of genotype E, genotype F also in 1962 (95% HPD: 51-62 years ago). Bayesian skyline analysis results showed that E6 fluctuated slightly in China during the years 2007-2009, especially circa 2009, and the population tended to expand on a small scale. After that, the effective population has remained at a stable level, and the change in virus mutation has always been at a low level (Fig. 3).

## Discussion

E6 is a major cause of outbreaks of AM, and the rising trend in the detection rate of E6 [32–34], indicates a great hidden danger to human health. Although China has not established systematic enterovirus surveillance, based on the surveillance of HFMD, we found that EVs other than non-EV-A71 and CV-A16 accounted for a high proportion of reported HFMD cases, particularly severe cases requiring medical attention. Most of the E6 sequences that were downloaded from GenBank for this study were from after 2000. This may indicate either that the surveillance of enteroviruses was strengthened during this period in different regions or that the activity of E6 in the population and environment has increased. At present, research on E6 around the world is limited to a single monitoring system in a localized area, and there is no uniform division of genotypes. Therefore, to reveal transmission networks and geographical distribution, it is of great importance to summarize the current data on global E6 and analyze its distribution using molecular methods.

Six genotypes were identified in this study, and the different genotypes were mainly associated with different diseases. Genotype C was mainly isolated from sewage for environmental monitoring, which indicates a potential risk of outbreaks. Although genotype E was prevalent among patients with AM/meningitis in the other countries, Chinese isolates of genotype E were more frequently detected in AFP patients, suggesting some genetic differences among the sequences in China and abroad. As the largest branch with the most extensive geographic distribution, genotype F includes viruses detected in China, Poland, Japan, Belarus, Finland, Ukraine, France, Tunisia, America, and Russia. These viruses were associated with diseases, including AM/meningitis, AFP, and HFMD, but were also found in healthy children from 1991 to 2018. Furthermore, more serious diseases were associated with genotype F than with the other genotypes; thus, we speculate that genotype F viruses have evolved with high transmissibility and virulence during persistent and extensive circulation among populations.

It is worth noting that in 1956 (95% confidence interval, 56-68 years ago), E6 gradually evolved into genotypes E and F and that circa 1962, different genotypes circulated independently. However, the sequences of the 1D and 3CD regions of 49 E6 strains in a geographical region in France from 1999 to 2007 were determined, and the substitution rate was estimated to be 8.597 × 10^-3^ and 6.252 × 10^-3^ substitutions site^-1^ year^-1^, respectively [35]. In our study, we included more sequences from over a wider range of time and area for evolutionary history analysis.

In the analysis of historical population dynamics, the E6 population in China showed a small shift in 2009 but did not cause large-scale disease outbreaks in the country. This phenomenon might be due to the evolution of E6 failing to reach a level that endangered human health or interference by other epidemic viruses. The detection rate of E6 in enterovirus and environmental monitoring is high [36], and recombination and mutations are the main determinants of viral sequence variation [37, 38]. This high degree of genetic diversity enables the virus to adapt quickly to the environment. Therefore, we conclude that the increase in the detection rate of the E6 strain is due to its rapid evolution.

## Conclusion

In this study, we pooled global E6 data and established a method for genotyping E6 using full-length VP1 sequences, which provides an important reference for E6 global sequence sharing and identification to determine how the virus evolved and where it originally emerged. Based on the analysis of inferred evolutionary trends, we calculated its evolution rate and estimated the time to the earliest common ancestor of all genotypes. Genotypes C and F were the predominant genotypes in China, and genotype F viruses might have evolved with high transmissibility and virulence during persistent and extensive circulation among populations.

This study also provides basic data for improving and enriching our understanding of the genetic characteristics and molecular epidemiology of E6. It is important to conduct continuous and extensive surveillance for E6. Further research on the evolution, transmissibility and virulence of this virus will provide essential data for evidence-based guiding disease control and treatment.

## Supplementary Information

Below is the link to the electronic supplementary material.

Supplementary file1 (DOC 126 KB)

## Data Availability

All data included in this study are available upon request by contacting the corresponding author.
