# Supplementary material for: Molecular epidemiological characteristics of echovirus 6 in mainland China: extensive circulation of genotype F from 2007 to 2018
Source: Arch Virol. 2021 Feb 26;166(5):1305–12. doi: 10.1007/s00705-020-04934-7 (PMC8036204; doi:10.1007/s00705-020-04934-7)
Supplement: Supplementary file 1 — Supplementary file1 (DOC 126 KB) [file 705_2020_4934_MOESM1_ESM.doc]

Molecular epidemiological characteristics of echovirus 6 in mainland China: genotype F circulated extensively in China from 2007 to 2018

Wenjun Cheng^1,2 #^, Tianjiao Ji^2#^, Shuaifeng Zhou^3^, Yong Shi^4^, Lili Jiang^5^, Yong Zhang^2^, Dongmei Yan^2^, Qian Yang^2^, Yang Song^2^, Ru Cai^1,*^, Wenbo Xu^1,2,6*^

**Supplementary Table 1.** Temporal and serotype distribution of EV isolates from the nationwide HFMD surveillance network in mainland China from 2007 to 2018

| Year | EV-A71 | CV-A16 | E6 | Other EV | Number of isolates | E6/Other EV (%) | E6/Number of isolates (%) |
| --- | --- | --- | --- | --- | --- | --- | --- |
| 2007* | 65 | 10 | 8 | 10 | 85 | 80.00 | 9.41 |
| 2008 | 236 | 94 | 0 | 34 | 389 | 0.00 | 0.00 |
| 2009 | 399 | 130 | 6 | 30 | 601 | 20.00 | 1.00 |
| 2010 | 1026 | 475 | 2 | 184 | 1644 | 1.09 | 0.12 |
| 2011 | 805 | 285 | 10 | 80 | 1204 | 12.50 | 0.83 |
| 2012 | 625 | 518 | 0 | 158 | 1288 | 0.00 | 0.00 |
| 2013 | 1022 | 541 | 59 | 1017 | 2490 | 5.80 | 2.37 |
| 2014 | 1203 | 1006 | 6 | 437 | 2618 | 1.37 | 0.23 |
| 2015 | 1069 | 850 | 4 | 797 | 2598 | 0.50 | 0.15 |
| 2016 | 975 | 768 | 1 | 819 | 2525 | 0.12 | 0.04 |
| 2017 | 1289 | 710 | 3 | 615 | 2881 | 0.49 | 0.10 |
| 2018 | 408 | 929 | 15 | 381 | 3216 | 3.94 | 0.47 |
| Total | 9122 | 6316 | 114 | 4562 | 21539 | 2.50 | 0.53 |

* As the HFMD surveillance system was established in mainland China in 2008, there is a certain bias regarding the 2007 data in the table.

**Supplementary Table 2.** The information for 87 E6 strains used for genotyping and 84 E6 strains used for bayesian evolutionary analysis.

| GenBank Number | Collection Country | Disease type | Collection Date | Disease severity | Origin |
| --- | --- | --- | --- | --- | --- |
| AB705310.1 | Japan | AM | 2011 | / | GenBank |
| EF397642.1 | Georgia | AM | 2005 | / | GenBank |
| EF397644.1 | Russia | AM | 2005 | / | GenBank |
| EF397650.1 | Ukraine | AM | 2006 | / | GenBank |
| EF397654.1 | Russia | AM | 2006 | / | GenBank |
| HQ399492.1 | China | Sewage | 2000 | / | GenBank |
| HQ399493.1 | China | Sewage | 2001 | / | GenBank |
| HQ399494.1 | China | Sewage | 2001 | / | GenBank |
| HQ399495.1 | China | Sewage | 2005 | / | GenBank |
| HQ674734.1 | Tunisia | Meningitis | 2001 | / | GenBank |
| HQ674736.1 | Tunisia | AFP | 1994 | / | GenBank |
| HQ674738.1 | Tunisia | AFP | 1993 | / | GenBank |
| HQ829951.1 | China | Sewage | 2010 | / | GenBank |
| KF487158.1 | China | Sewage | 2009 | / | GenBank |
| KF487190.1 | China | Sewage | 2011 | / | GenBank |
| KF487201.1 | China | Sewage | 2012 | / | GenBank |
| KJ754036.1 | China | AFP | 2010 | / | GenBank |
| KJ754037.1 | China | AFP | 2008 | / | GenBank |
| KJ754038.1 | China | AFP | 2007 | / | GenBank |
| KJ772446.1 | China | AFP | 2000 | / | GenBank |
| KJ772462.1 | China | AFP | 2007 | / | GenBank |
| KJ772479.1 | China | AFP | 2011 | / | GenBank |
| KJ772482.1 | China | AFP | 1988 | / | GenBank |
| KJ772484.1 | China | AFP | 1993 | / | GenBank |
| KJ772487.1 | China | AFP | 1995 | / | GenBank |
| KJ772490.1 | China | AFP | 1997 | / | GenBank |
| KJ772492.1 | China | AFP | 1998 | / | GenBank |
| KJ772493.1 | China | AFP | 1999 | / | GenBank |
| KM624568.1 | China | Sewage | 2013 | / | GenBank |
| KT633557.1 | China | AM | 2013 | / | GenBank |
| KX867977.1 | China | Sewage | 2015 | / | GenBank |
| KY048060.1 | China | AM | 2014 | / | GenBank |
| LC167432.1 | China | HC | 2016 | / | GenBank |
| LN713456.1 | Tunisia | Meningitis | 2011 | / | GenBank |
| MF467363.1 | China | AM | 2015 | / | GenBank |
| MK086231.1 | France | Sewage | 2015 | / | GenBank |
| MK570391.1 | Brazil | AM | 2013 | / | GenBank |
| MK570398.1 | Brazil | AM | 2014 | / | GenBank |
| MK570437.1 | Brazil | AM | 2016 | / | GenBank |
| MK570450.1 | Brazil | AM | 2017 | / | GenBank |
| JN203704.1 | India | / | / | / | GenBank |
| JN203711.1 | India | / | / | / | GenBank |
| JQ929657.2 | Finland | / | 2009 | / | GenBank |
| KF177018.1 | India | / | 2009 | / | GenBank |
| KF177021.1 | India | / | 2010 | / | GenBank |
| KX683317.1 | Poland | / | 2014 | / | GenBank |
| KX683340.1 | Poland | / | 2012 | / | GenBank |
| KX683351.1 | Poland | / | 2012 | / | GenBank |
| FJ868295.1 | Australia | / | 1991 | / | GenBank |
| FJ868317.1 | Australia | / | 1991 | / | GenBank |
| FN688503.1 | France | / | 2000 | / | GenBank |
| FN688520.1 | France | / | 2002 | / | GenBank |
| FN688528.1 | France | / | 2004 | / | GenBank |
| GQ329779.1 | China | HFMD | 2002 | / | GenBank |
| GQ329780.1 | China | HFMD | 2006 | / | GenBank |
| GQ329781.1 | China | HFMD | 2008 | / | GenBank |
| GQ329784.1 | China | HFMD | 1998 | / | GenBank |
| GQ352380.1 | Belarus | / | 2003 | / | GenBank |
| GU142887.1 | Australia | / | 1999 | / | GenBank |
| KR232693.1 | Madagascar | / | 2011 | / | GenBank |
| AF081321.1 | / | / | 1955 | / | GenBank |
| AF081322.1 | / | / | / | / | GenBank |
| AF081323.1 | / | / | 1957 | / | GenBank |
| NX11-143 | [Northwest China](http://www.baidu.com/link?url=PKA8G_a2lSZyF5sRT73MQSWvoxt-lyY-DNVqzHuVTNl9cg2mO-Ir7fGNFvl75qWbXUV2uxYtcuhg9YS9tPr7n2IQk3K5c06KzxpJuo2tJPIdo60a9aQjOJAu7eePBl8-) | HFMD | 2011 | fatal case | This study |
| HeB15-54497H | North China | HFMD | 2015 | mild case | This study |
| SD11-133 | East China | HFMD | 2011 | mild case | This study |
| JL17-27 | Northeast China | HFMD | 2017 | mild case | This study |
| SC13-66 | Southwest China | HFMD | 2013 | mild case | This study |
| YN09-T261 |  | HFMD | 2009 | severe case | This study |
| YN16-A6 |  | HFMD | 2016 | mild case | This study |
| CQ13-39 |  | HFMD | 2013 | mild case | This study |
| GX18-350 |  | HFMD | 2018 | severe case | This study |
| GY18 |  | HFMD | 2018 | mild case | This study |
| GZ13-6 |  | HFMD | 2013 | mild case | This study |
| HeN13-337 | Central China | HFMD | 2013 | mild case | This study |
| HeN13-898 |  | HFMD | 2013 | severe case | This study |
| HUN09-25 |  | HFMD | 2009 | severe case | This study |
| HUN10-99 |  | HFMD | 2010 | severe case | This study |
| HUN13-34 |  | HFMD | 2013 | severe case | This study |
| HUN14-34 |  | HFMD | 2014 | mild case | This study |
| HUN14-39 |  | HFMD | 2014 | mild case | This study |
| HUN14-70 |  | HFMD | 2014 | mild case | This study |
| JS13-119 | East China | HFMD | 2013 | severe case | This study |
| JX15-110 |  | HFMD | 2015 | mild case | This study |
| JX15-27 |  | HFMD | 2015 | mild case | This study |
| JX18-106 |  | HFMD | 2018 | mild case | This study |
| JX18-87 |  | HFMD | 2018 | mild case | This study |

**Supplementary Table 3.** The information about the sequences downloaded from GenBank used in this study.

| Disease type | Number of Sequences (percentage) | Country | Year |
| --- | --- | --- | --- |
| AM/Meningitis | 271(38.28%) | Russia/China/France/Kyrgyzstan Georgia/Ukraine/Azerbaijan/Belarus/Algeria/Poland/Brazil/Tunisia/Japan | 1999-2017 |
| HFMD | 18(2.54%) | China | 1998-2014 |
| AFP | 92(12.99%) | China/India/Niger | 1988-2014 |
| Sewage | 176(24.86%) | France/China/Poland/Haiti | 2000-2016 |
| Healthy children | 17(2.40%) | China | 2013-2016 |
| Unknown | 134(20.20%) | Netherlands/Australia/Madagascar/Senegal/China/France | 1955-2016 |
| Total | 708^*^ | 5 continents and 20 countries | 1955-2017 |

AM: aseptic meningitis; HFMD：[hand foot and mouth](javascript:;) [disease](javascript:;); AFP: acute flaccid paralysis

**Supplementary Table 4.** The distribution of time about the sequences of E6 in this study.

| Year | Number of Sequences  (percentage) |
| --- | --- |
| 1955-1999 | 35(4.26%) |
| 2000-2007 | 203(24.70%) |
| 2008-2013 | 377(45.86%) |
| 2014-2018 | 190(23.11%) |
| unknown | 17(2.01%) |
| Total | 822 |
